# Supplementary material for: Dissecting the Activity of Catechins as Incomplete Aldose Reductase Differential Inhibitors through Kinetic and Computational Approaches
Source: Biology (Basel). 2022 Sep 6;11(9):1324. doi: 10.3390/biology11091324 (PMC9495972; doi:10.3390/biology11091324)
Supplement: Supplementary file 1 [file biology-11-01324-s001.zip › biology-1882096-supplementary.pdf]

Supplementary Materials

DIFFERENTIAL INHIBITION OF ALDOSE REDUCTASE BY CATHECHINS

Table S1. Binding free energy and average ligand RMSD values calculated for the ten AKR1B1-L-idose-GCG complexes analyzed through MD simulations.

**Complex**	**ΔPBSA (kcal/mol)**	**Ligand RMSD (Å)**
**1**	-25.2	2.26
**2**	-18.1	5.98
**3**	-9.5	8.76
**4**	-13.5	2.02
**5**	-17.5	1.44
**6**	-7.3	4.45
**7**	-9.5	6.50
**8**	-16.0	4.63
**9**	-8.0	3.96
**10**	-10.6	8.51

Table S2. Binding free energy and average ligand RMSD values calculated for the nine AKR1B1-GCG complexes (closed specificity pocket conformation) analyzed through MD simulations.

Complex	ΔPBSA (kcal/mol)	Ligand RMSD (Å)
**1**	-20.2	1.16
**2**	-24.2	0.88
**3**	-15.4	3.10
**4**	-14.5	5.76
**5**	-20.3	2.70
**6**	-22.8	3.83
**7**	-19.0	3.52
**8**	-23.7	1.85
**9**	-17.6	2.95

Table S3. Binding free energy and average ligand RMSD values calculated for the four AKR1B1-GCG complexes (open specificity pocket conformation) analyzed through MD simulations.

Complex	ΔPBSA (kcal/mol)	Ligand RMSD (Å)
**1**	-12.1	2.39
**2**	-12.9	3.64
**3**	-14.0	3.37
**4**	-19.4	3.22

Table S4. Binding free energy and average ligand RMSD values calculated for the ten AKR1B1-HNE-GCG complexes analyzed through MD simulations.

Complex	ΔPBSA (kcal/mol)	Ligand RMSD (Å)
**1**	-23.3	1.29
**2**	-18.4	1.54
**3**	-18.8	2.16
**4**	-13.1	8.51
**5**	-14.4	2.54
**6**	-22.7	7.08
**7**	-10.9	5.84
**8**	-11.9	5.08
**9**	-10.4	7.88
**10**	-5.7	3.08

Table S5. Binding free energy and average ligand RMSD values calculated for the ten AKR1B1-GSHNE-GCG complexes analyzed through MD simulations.

Complex	ΔPBSA (kcal/mol)	Ligand RMSD (Å)
**1**	-8.0	3.73
**2**	-13.1	4.48
**3**	-15.0	2.31
**4**	-13.8	5.13
**5**	-25.5	2.04
**6**	-7.5	2.48
**7**	-16.5	4.64
**8**	-10.1	3.97
**9**	-12.8	4.70
**10**	-13.9	4.97
